# Identification of unique ROH regions with unfavorable effects on production and fertility traits in Canadian Holsteins

**DOI:** 10.1186/s12711-021-00660-z

**Published:** 2021-08-30

**Authors:** Bayode O. Makanjuola, Christian Maltecca, Filippo Miglior, Gabriele Marras, Emhimad A. Abdalla, Flavio S. Schenkel, Christine F. Baes

**Affiliations:** 1 Centre for Genomic Improvement of Livestock, Department of Animal Biosciences, University of Guelph, Guelph, ON N1G 2W1 Canada; 2 Department of Animal Science and Genetics Program, North Carolina State University, Raleigh, NC 27607 USA; 3 Semex Alliance, Guelph, ON N1H 6J2 Canada; 4 Institute of Genetics, Vetsuisse Faculty, University of Bern, 3001 Bern, Switzerland

## Abstract

**Background:**

The advent of genomic information and the reduction in the cost of genotyping have led to the use of genomic information to estimate genomic inbreeding as an alternative to pedigree inbreeding. Using genomic measures, effects of genomic inbreeding on production and fertility traits have been observed. However, there have been limited studies on the specific genomic regions causing the observed negative association with the trait of interest. Our aim was to identify unique run of homozygosity (ROH) genotypes present within a given genomic window that display negative associations with production and fertility traits and to quantify the effects of these identified ROH genotypes.

**Methods:**

In total, 50,575 genotypes based on a 50K single nucleotide polymorphism (SNP) array and 259,871 pedigree records were available. Of these 50,575 genotypes, 46,430 cows with phenotypic records for production and fertility traits and having a first calving date between 2008 and 2018 were available. Unique ROH genotypes identified using a sliding-window approach were fitted into an animal mixed model as fixed effects to determine their effect on production and fertility traits.

**Results:**

In total, 133 and 34 unique ROH genotypes with unfavorable effects were identified for production and fertility traits, respectively, at a 1% genome-wise false discovery rate. Most of these ROH regions were located on bovine chromosomes 8, 13, 14 and 19 for both production and fertility traits. For production traits, the average of all the unfavorably identified unique ROH genotypes effects were estimated to decrease milk yield by 247.30 kg, fat yield by 11.46 kg and protein yield by 8.11 kg. Similarly, for fertility traits, an average 4.81-day extension in first service to conception, a 0.16 increase in number of services, and a − 0.07 incidence in 56-day non-return rate were observed. Furthermore, a ROH region located on bovine chromosome 19 was identified that, when homozygous, had a negative effect on production traits. Signatures of selection proximate to this region have implicated *GH1* as a potential candidate gene, which encodes the growth hormone that binds the growth hormone receptor. This observed negative effect could be a consequence of unfavorable alleles in linkage disequilibrium with favorable alleles.

**Conclusions:**

ROH genotypes with unfavorable effects on production and fertility traits were identified within and across multiple traits on most chromosomes. These identified ROH genotypes could be included in mate selection programs to minimize their frequency in future generations.

**Supplementary Information:**

The online version contains supplementary material available at 10.1186/s12711-021-00660-z.

## Background

The profitability of dairy cattle production depends on production output, reproduction and overall health and fitness of the animals. However, these characteristics could be negatively affected by inbreeding depression, which often arises as a consequence of increasing levels of inbreeding. Inbreeding depression can occur as a result of increased deleterious recessive homozygous alleles, thereby causing reduction of fitness and production and an increase in the incidence of lethal or harmful defects. Some identified examples of lethal defects are: bovine leukocyte adhesion (BLAD) [1], deficiency of urine monophosphate synthase (DUMPS) [2] and complex vertebral malformation (CVM) [3]. In addition, highly inbred offspring often have decreased mean phenotypic values for traits associated with overall fitness in a given population [4]. With the growing interest in the advancement and implementation of genomic selection, North American Holstein breeds have witnessed a greater than twofold increase in the rate of inbreeding per year, thus substantially reducing their effective population size [5]. This decline in the effective population size could lead to higher genetic relationships between animals, therefore further increasing the frequency of deleterious recessive homozygous mutations and ultimately causing inbreeding depression within the population. Economic losses associated with inbreeding depression have also been documented, e.g. Croquet et al. [6] reported that for every 1% increase in inbreeding of the Walloon Belgian Holstein cattle, a loss of 6.13 euros in their global economic index for lifetime profit occurs. Similarly, approximately 11 million dollars could potentially be lost by an increased frequency of deleterious recessive haplotypes from the mating of carrier individuals in the US dairy population [7]. Fleming and Van Doormaal [8] recently reported that the breakeven age of Holstein cows in Canada, which is the profit recovered after removing the cost incurred from rearing the cows, is largely determined by the age at first calving and milk production. However, if these traits are depressed because of rising inbreeding levels, the cost of rearing inbred cows will outweigh the total revenue obtained.

Various authors have investigated the effect of inbreeding on traits that are routinely measured in animals for genetic evaluations (for a detailed review see [9, 10]). This has been traditionally performed by regressing the trait of interest on pedigree estimated inbreeding coefficients. Based on pedigree data, inbreeding has been found to have an unfavorable effect on milk production traits [6, 11–13], fertility [13–15], and survival [16–18]. With the availability of genomic information, the effects of inbreeding have been estimated by using genomic inbreeding coefficients. Using this measure, adverse effects of inbreeding were reported for economically important traits [19–21]. Of particular interest, is the occurrence of runs of homozygosity (ROH), which are unbroken homozygous genomic segments that are present on a given chromosome of an individual and that have been proposed as a better indicator of inbreeding because they show higher correlations with deleterious mutation load [22]. In addition, based on a simulation study reported by Forutan et al. [23], inbreeding coefficients estimated from ROH are closer to the true inbreeding coefficient estimates. Furthermore, inbreeding coefficients can be estimated at the chromosomal level with a heterogeneous distribution observed along the chromosomes [24, 25], making ROH suitable for identifying chromosomal regions that have a negative effect on economically important traits. Pryce et al. [19] found a ROH region on *Bos taurus* (BTA) chromosome 20 in both Holsteins and Jerseys that was negatively associated with milk yield, causing a reduction of 161 and 194 L in milk yield per lactation, respectively. In addition, Martikainen et al. [25] identified a region on BTA2 in Finnish Ayrshires with an unfavorable effect on the interval from first to last insemination on heifers, thereby lengthening the time period for heifers carrying this region by ~ 1.6 days. The approach used in these previous studies was simply to estimate the effect of a region located within a ROH on phenotypes of interest. However, this approach does not directly identify the different unique ROH genotypes within a genomic region that leads to the unfavorable effect, which results in the observed reduced performances. Therefore, the objectives of our study were to (1) identify localized chromosomal regions that are negatively associated with production and fertility traits; and (2) identify unique ROH genotypes with unfavorable effects within and across multiple traits.

## Methods

### Pedigree data

A pedigree file including 259,871 Holstein animals was made available by the Canadian Dairy Network (CDN, partner of the Lactanet group) with a base year population set at 1950. Therefore, animals in this base year were assumed to be unrelated. We used the pedigree information that was relevant to all the animals with phenotypic and genotypic data in the analyses.

### Genotype data

Genotypes of 50,575 Holstein cows were available with birth year ranging from 1999 to 2017. Of these 50,575 cows, ~ 34% were genotyped with the Illumina BovineSNP50 Chip (50K) (Illumina Inc., San Diego) and ~ 66% were genotyped with lower-density array panels. For the low-density panels, the number of available SNPs ranged from 7 to 30K SNPs. Animals with lower-density genotypes were imputed to medium-density (50K) using the FImpute software [26]. The accuracy of imputed genotypes was on average 99% (allelic r^2^) attributable to the use of pedigree information and of a large number of reference animals (50,000 key ancestors) with 50K genotypes. Before editing, SNP information was available for 45,187 SNPs. The SNP positions for the genotypes used were based on the most recent bovine genome assembly ARS-UCD 1.2. For quality control, only autosomal SNPs with a call rate higher than 0.95, a minor allele frequency higher than 0.01 and a difference between observed and expected heterozygosity frequency smaller than 0.15 were retained for further analyses using the SNP1101 package [27]. After quality control, 43,126 SNPs remained.

### Phenotype data

Phenotypic records on 46,430 first lactation genotyped cows with first calving records obtained from 2008 to 2018 were available for production and fertility traits. The production traits available for this study included milk yield in kg (MY), fat yield in kg (FY) and protein yield in kg (PY) on 21,194 cows with first lactation records on a 305-day basis. In total, 33,610 first lactation cows with fertility traits such as: age at first service in days (AFS), number of services (NS), first service non-return rate to 56 days (NRR) and days from first service to conception (FSTC) were available. The NRR fertility trait was coded 1 when no subsequent service took place between 15 and 56 days following the first service, and coded 0 otherwise. NS was coded from 1 to 10, and animals with more than 10 services were assigned as 10. AFS and FSTC were measured in days. A summary of the analyzed traits is in Table 1.

**Table 1:** Descriptive statistics of the analyzed traits, including number of records, mean, standard deviation (SD), minimum (min) and maximum (max) number of observations

Traits	Number of records	Mean	SD	Min	Max
MY (kg)	21,194	9074	1732.22	1140	17,542
FY (kg)	21,194	362	73.85	39	919
PY (kg)	21,194	295.50	54.58	39	603
AFS (day)	33,610	449.20	49.16	274	639
NS	33,610	1.59	0.93	1	7
NRR	33,610	0.69	0.46	0	1
FSTC (day)	33,610	19.32	33.37	0	205

MY: milk yield; FY: fat yield; PY: protein yield; AFS: age at first service; NS: number of services; NRR: 56-day non-return rate; FSTC: first service to conception

It is worth noting that cows with production records also had fertility records, and that the number of production records was smaller than that of fertility records because of partial lactation.

### Statistical analyses

Unique ROH genotypes with unfavorable effects on phenotypes were identified using the algorithm developed by Howard et al. [28], which has been described in detail as *Haplofinder* (see [28]). Briefly, the algorithm uses a sliding window approach and the procedure for the identification of unfavorable haplotypes is divided into three steps. In the first step (1), a window size of a predetermined number of SNPs (default = 60) starting from the first SNP of a chromosome is constructed. Within a window, each unique ROH genotype with an unfavorable effect on phenotypes is identified when a minimum of 15 consecutive homozygous SNPs with no heterozygous genotypes allowed within the ROH genotypes and the frequency of each unique ROH genotype is higher than 0.75%. This means that ROH genotypes with heterozygous genotypes and a frequency lower than 0.75% were categorized as non-ROH classes. Then, the phenotypic mean was estimated for each unique ROH genotype using all the individuals carrying the unique ROH genotype. The windows containing ROH genotypes with a phenotypic mean value above or below a user-defined threshold depending on the unfavorable direction were stored for further analyses. The procedure used to determine this threshold is described in more detail later in this section. After storing, the window was moved forward along the chromosome by steps of one SNP and the previously explained process was repeated, until the entire chromosome was scanned for the identification of unique ROH genotypes. After scanning the entire chromosome, windows with the same set of animals carrying the same set of unique ROH genotypes except for the first and last SNP were aggregated. Subsequently, the length of the window was further reduced by five SNPs and the previous steps were repeated using this new window size. This reduction in window size was continued until a minimum window size of 50 SNPs was reached. The criterion to retain a window size with a minimum of 50 SNPs was based on results in the literature that suggest that such a window size captures the more recent inbreeding [19] since recent inbreeding is known to exhibit more detrimental effects on the phenotype than ancient inbreeding [13, 29]. Therefore, only ROH genotypes with a minimum of 50 SNPs were retained.

After identification of all the unique unfavorable ROH genotypes in step (1), the second step (2) consisted in testing the significance of the effects of all remaining windows on the phenotypes of interest by fitting the identified unique ROH genotype and non-ROH for a given window as a fixed class effect along with other fixed effects and random effects in an animal mixed model using the model employed for the national genetic evaluation of Canadian Holsteins [30]. Because the aim of our study was to estimate the effect of each unique unfavorable ROH genotype, the model also fitted the SNPs present in the unfavorable ROH genotypes to correct for their additive effects. The other fixed effects were: year of calving by season of calving (YSC); age at calving by region of calving (ARC); region by year of birth by season of birth (RYS); month of first insemination (Mf); age at previous calving by month of previous calving by parity (ApMp); and age at previous calving by month of first insemination (ApMf). The random effects were: herd by year of birth (HY); herd within RYS (HRYS); service sire by year of insemination (SS); artificial insemination technician (AIT); animal additive genetic effect (A); and an error term (E). Each trait was evaluated separately to estimate the effect of the identified unique ROH genotypes using the following specific linear mixed model:

$${\mathbf{y}} = {\mathbf{Xb}} + {\mathbf{Za}} + \mathop \sum \limits_{{{\varvec{j}} = 1}}^{{\varvec{n}}} {\mathbf{Wc}}_{{\mathbf{j}}} + {\mathbf{e}},$$

where ${\mathbf{y}}$ is a vector of phenotypic measurements for MY, FY, PY, AFS, NS, NRR and FSTC, ${\mathbf{b}}$ is a vector of systematic fixed effects as described above, as well as the ROH genotypes in a given window, ${\mathbf{a}}$ is a vector of random additive genetic effects, ${\mathbf{c}}_{{\mathbf{j}}}$ is the ${\text{j}}$-*th* non-genetic random effect, ${\mathbf{e}}$ is a vector of the random error terms, $n$ is the number of non-genetic random effects (4 effects, as defined above), ${\mathbf{X}}$, ${\mathbf{Z}}$ and ${\mathbf{W}}$ are design matrices that relate fixed effects, random additive genetic effects and non-genetic random effects to the phenotype, respectively. The assumptions for the random effects included: ${\mathbf{a}} \sim {\text{N}}\left( {0, {\mathbf{A}}{\upsigma }_{{\text{a}}}^{2} } \right)$, ${\text{HY}} \sim {\text{N}}\left( {0, {\mathbf{I}}{\upsigma }_{{{\text{HY}}}}^{2} } \right)$, ${\text{HRYS}} \sim {\text{N}}\left( {0, {\mathbf{I}}{\upsigma }_{{{\text{HRYS}}}}^{2} } \right)$, ${\text{T}} \sim {\text{N}}\left( {0, {\mathbf{I}}{\upsigma }_{{{\text{AIT}}}}^{2} } \right)$, ${\text{SS}} \sim {\text{N}}\left( {0, {\mathbf{I}}{\upsigma }_{{{\text{SS}}}}^{2} } \right)$ and ${\mathbf{e}} \sim {\text{N}}\left( {0, {\mathbf{I}}{\upsigma }_{{\text{e}}}^{2} } \right)$, where ${\upsigma }_{{\text{a}}}^{2}$ is the additive genetic variance, ${\upsigma }_{{{\text{HY}}}}^{2}$ is the herd year variance, ${\upsigma }_{{{\text{HRYS}}}}^{2}$ is the HRYS variance, ${\upsigma }_{{{\text{SS}}}}^{2}$ is the service sire by year of insemination variance, ${\upsigma }_{{{\text{AIT}}}}^{2}$ is the artificial insemination technician variance, $\sigma_{e}^{2}$ is the residual variance, ${\mathbf{A}}$ is the numerator relationship matrix and ${\mathbf{I}}$ is an identity matrix. The variance components of this implementation were assumed fixed across windows based on the null hypothesis of no ROH effect. Given the solutions for each window, the contrast between each unique ROH genotype and non-ROH genotype along with their respective *t*-statistics was obtained. To control the false discovery rate from multiple testing and to identify ROH genotypes with significant effects on phenotypes, the genome-wise false discovery rate (FDR) was controlled at 1%, using the method proposed by Benjamini and Hochberg [31]. Given that a minimum threshold value was defined and only ROH genotypes with unfavorable phenotypic means were selected (above or below the threshold, depending on the trait), the null hypothesis indicated that no differences between ROH genotypes and non-ROH genotypes existed; and the alternate hypothesis signifies that a difference between ROH genotypes and non-ROH genotypes is observed. The hypothesis test is a one-tailed *t-test*, which considers only the unfavorable direction of the ROH genotypes, thus, animals that carry non-ROH genotypes are assumed to be the benchmark for the comparison with animals that carry the ROH genotypes. A significant effect of the regression coefficients estimated from the mixed model indicates that there is a difference between the mean phenotypic value of animals that carry the ROH genotypes and those that carry the non-ROH genotypes. Using this parameterization to estimate inbreeding depression aligns with the partial dominance hypothesis, which has been reported to account for most of the inbreeding depression observed within a given population [32, 33]. Furthermore, the justification of using unique ROH genotypes within a window over traditional single SNP or marker haplotypes is that ROH have been shown to have higher correlations with deleterious mutation load [22], as well as having inbreeding estimates closer to true inbreeding [23]. Finally, the third step (3) involves the window reduction step, whereby windows that contain the same set of animals nested within another window are discarded.

For estimating the user-defined threshold used to determine the unfavorable direction, a cut-off value for the mean phenotype considered unfavorable was generated by an empirical *t*-statistic distribution from the available phenotypic data. This was performed by randomly sampling windows across the genome and estimating the statistical significance of the ROH genotypes present in the window. The random sampling of windows was repeated 1000 times to generate statistically significance levels. Across samples, a cut-off value was selected based on the average phenotype for *t*-statistics with a significance that ranged from 0.05 to 0.10. Thereafter, the direction of the unfavorable effect was determined based on whether the mean phenotype of the individuals that carry a unique ROH genotype in a given window is below or above the cut-off value depending on the trait. For example, the unfavorable direction of a trait such as MY was determined when the mean phenotype of individuals with a unique ROH genotype in a given window was less than the cut-off value. The *Haplofinder* software and its source code can be accessed from https://github.com/jeremyhoward.

### Functional analyses

Each unique ROH genotype with a significant unfavorable effect on phenotype based on an FDR of 1% was further investigated to identify potential candidate genes that could be involved in the observed detrimental effects. Annotated genes located within the significant ROH genotypes were obtained from the Ensembl BioMart Martview (http://useast.ensembl.org/biomart/martview/) using the new bovine genome assembly ARS-UCD 1.2 (release 99).

## Results

### Chromosomal regions associated with production traits

**Fig. 1:**
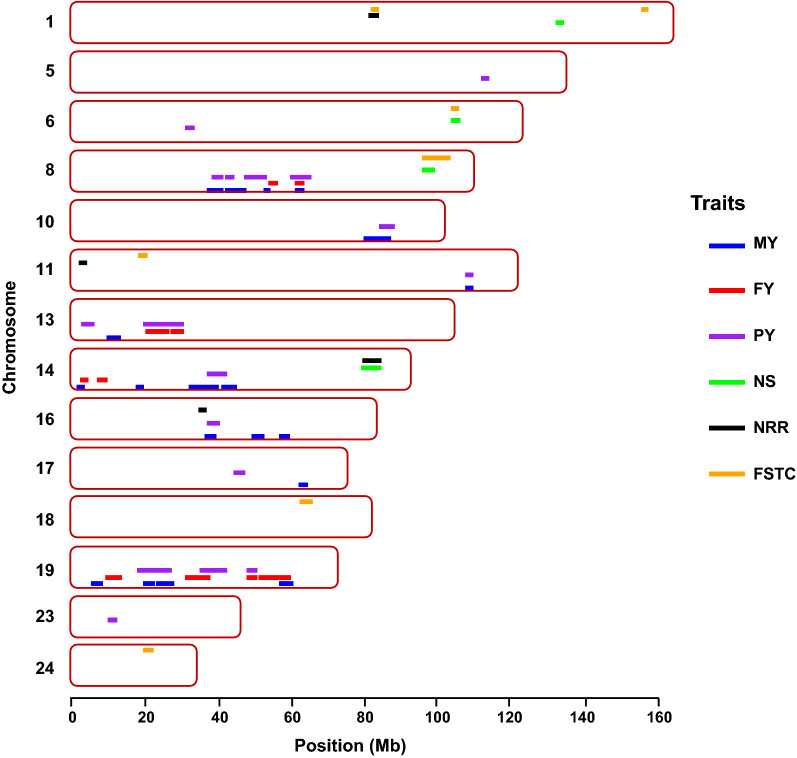
Unfavorable ROH genotypes within chromosomes identified within and across multiple traits. MY: milk yield; FY: fat yield; PY: protein yield; NS: number of services for heifers; NRR: 56-day non-return rate for heifers; FSTC: first service to conception for heifers

**Fig. 2:**
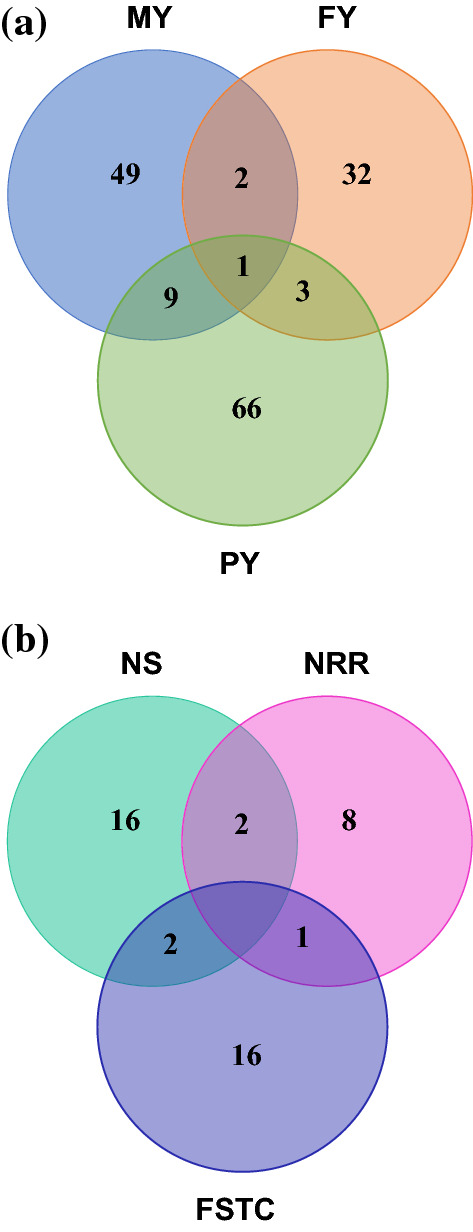
A Venn diagram showing the number of unique ROH genotypes identified within and between production traits (**a**) and within and between fertility traits (**b**). MY: milk yield; FY: fat yield; PY: protein yield; NS: number of services for heifers; NRR: 56-day non-return rate for heifers; FSTC: first service to conception for heifers

ROH genotypes unfavorably associated with production traits were identified on almost all the chromosomes (Fig. 1), but not all chromosomes harboured regions that negatively affected all the traits. More specifically, ROH regions with unfavorable effects were observed on BTA8, 10, 11, 13, 14, 16, 17 and 19 for MY; BTA8, 13, 14 and 19 for FY; and BTA5, 6, 8, 10, 11, 13, 14, 16, 17, 19 and 23 for PY. For the production traits, the average lengths of the ROH with unfavorable effects were 2.59, 2.87 and 2.71 Mb for MY, FY and PY, respectively (Table 2). In total, 49, 32 and 66 ROH regions with negative effects on MY, FY and PY were identified, respectively (Fig. 2a).

**Table 2:** Summary statistics showing the average, minimum and maximum length and the average number of SNPs within the uniquely identified ROH with unfavorable effects on production and fertility traits

Traits	Average number of SNPs	Average	Minimum	Maximum
MY (kg)	50.43	2.59	1.94	3.68
FY (kg)	50.38	2.87	1.70	4.06
PY (kg)	50.33	2.71	1.89	4.07
NS	50.50	2.61	1.94	3.40
NRR	50.50	2.41	1.75	3.40
FSTC (day)	50.19	2.58	2.09	3.39

MY: milk yield; FY: fat yield; PY: protein yield; NS: number of services; NRR: 56-day non-return rate; FSTC: first service to conception

The most extreme ROH genotypes (i.e. ROH regions showing the largest unfavorable effect per trait) were found on BTA14 for both MY and FY and on BTA5 for PY (Table 3). Furthermore, animals carrying the most extreme ROH genotype on BTA14 for MY and FY had on average 410.65 and 15.81 kg less MY and FY per lactation, respectively, than animals that did not carry the ROH genotype. Similarly, a reduction of 16.12 kg in PY was observed for animals with the most extreme ROH genotype on BTA5 compared to those with non-ROH genotypes.

**Table 3:** Significant unique ROH regions with the most extreme unfavorable effects on production and fertility traits

Trait	BTA	Start pos (Mb)	End pos (Mb)	ROH effects^a^	−log_10_ (P value)
MY (kg)	14	16.49	18.88	− 410.65	4.91
FY (kg)	14	1.99	4.05	− 15.81	5.31
PY (kg)	5	107.61	109.73	− 16.12	6.45
NS	6	99.80	102.15	0.23	5.44
NRR	14	78.31	81.47	− 0.10	3.55
FSTC (day)	6	99.78	102.02	7.87	4.86

MY: milk yield; FY: fat yield; PY: protein yield; NS: number of services; NRR: 56-day non-return rate; FSTC: first service to conception; BTA: *Bos taurus* chromosome

^a^Effects of uniquely identified ROH in comparison to the non-ROH class

### Chromosomal regions associated with fertility traits

ROH genotypes with detrimental effects were localized and identified for three of the four fertility traits in our study. Based on the criteria used, no ROH genotype with a significant unfavorable effect on AFS was identified (Fig. 1). The length of the ROH genotypes identified with unfavorable effects on fertility traits ranged from 1.75 to 3.40 Mb (Table 2). The total number of observed ROH genotypes with an unfavorable effect was equal to 16, 8 and 16 for NS, NRR and FSTC, respectively (Fig. 2b). Unfavorable ROH genotypes were identified on BTA1, 6, 8 and 14 for NS, on BTA1, 11, 14 and 16 for NRR, and on BTA1, 6, 8, 11, 18 and 24 for FSTC (Fig. 1). The most extreme ROH region with an unfavorable effect on FSTC was found on BTA6, i.e. a 7.87-day extension of conception following first service was observed for animals that carry the ROH genotype compared to animals that did not carry it. Likewise, we observed the most extreme ROH genotype on BTA6 for NS, which led to a 0.23 increased chance of having more inseminations following the first insemination. In addition, the most extreme ROH genotype with an unfavorable effect on NRR was found on BTA14, with the animals that carry this ROH genotype presenting a 10% higher incidence of having a subsequent service between 15 and 56 days after the first service than the animals that did not carry it.

### Chromosomal regions associated with two or more traits

Pleiotropic effects of some unfavorable ROH genotypes were identified across multiple traits. The numbers of ROH genotypes showing pleiotropic effects are shown in Fig. 2a and b. Given that a genome-wise FDR of 1% was used, we found no overlapping ROH genotype with an unfavorable effect between production and fertility traits. Therefore, ROH genotypes with pleiotropic effects were categorized into two groups: (1) ROH genotypes with unfavorable effects on production traits; and (2) ROH genotypes with unfavorable effects on fertility traits. Only one ROH genotype had a significant unfavorable effect on all production traits. In total, we identified three ROH genotypes affecting MY and FY, four ROH genotypes affecting FY and PY, and two ROH genotypes affecting NS and FSTC. For production traits, the unfavorable ROH genotype on BTA8 had a − 364.23 kg, − 11.69 kg and − 11.06 kg effect on MY, FY and PY, respectively. For fertility traits, the unfavorable ROH genotype identified on BTA1 caused a − 0.41 decrease in NRR and a 2.78-day increase in FSTC. All ROH genotypes showing significant pleiotropic unfavorable associations with multiple traits are in Table 4.

**Table 4:** Significant unique ROH genotypes with unfavorable effects on multiple traits

BTA	Trait	Interval (Mb)	ROH effects^a^	Frequency of ROH genotypes (%)	−log_10_ (P value)
8	MY (kg)	58.61–61.11	− 364.23	1.55	6.50
	FY (kg)		− 11.69		4.54
	PY (kg)		− 11.06		6.26
19	MY (kg)	54.48–56.53	− 280.18	1.66	4.78
	FY (kg)		− 14.23		6.07
10	MY (kg)	81.29–84.14	− 297.84	1.44	5.19
	PY (kg)		− 9.96		4.77
11	MY (kg)	103.48–105.76	− 252.69	2.44	5.36
	PY (kg)		− 8.35		5.78
14	MY (kg)	35.43–37.93	− 192.90	3.86	5.01
	PY (kg)		− 5.84		4.82
19	MY (kg)	18.51–21.50	− 225.30	2.51	4.71
	PY (kg)		− 7.84		5.40
19	MY (kg)	22.00–24.50	− 205.46	2.86	4.54
	PY (kg)		− 7.06		5.12
19	MY (kg)	23.71–25.85	− 205.96	2.93	4.62
	PY (kg)		− 7.08		5.21
19	MY (kg)	23.73–25.91	− 215.11	2.97	4.92
	PY (kg)		− 7.22		5.40
19	MY (kg)	23.81–25.97	− 218.09	2.95	4.98
	PY (kg)		− 7.36		5.50
13	FY (kg)	21.78–25.44	− 12.10	2.02	5.56
	PY (kg)		− 8.22		4.99
19	FY (kg)	45.84–48.66	− 9.53	2.01	4.11
	PY (kg)		− 6.33		3.64
14	NS	76.45–79.85	0.19	0.78	3.43
	NRR		− 0.09		2.91
14	NS	78.31–81.47	0.19	0.76	3.25
	NRR		− 0.10		3.55
1	NRR	78.55–80.97	− 0.41	4.25	3.32
	FSTC (day)		2.78		2.96
8	NS	92.77–95.67	0.16	1.30	3.60
	FSTC (day)		5.59		3.58
6	NS	99.75–102.02	0.23	0.97	5.35
	FSTC (day)		7.87		4.86

MY: milk yield; FY: fat yield; PY: protein yield; NS: number of services; NRR: 56-day non-return rate; FSTC: first service to conception; BTA: *Bos taurus* chromosome

^a^Effects of uniquely identified ROH in comparison to the non-ROH class

### Potential candidate genes associated with production and fertility traits

We investigated the presence of potential candidate genes known to impact production and fertility traits (see Additional file 1: Table S1) in the identified ROH genotypes with significant unfavorable effects by identifying all the genes they harbored and analysing their biological and molecular pathways. The most interesting genes are those located within ROH genotypes that have effects on multiple traits (Table 5) because they may be more sensitive to inbreeding and consequently cause a more robust reduction in the overall fitness of the individual. We detected two candidate genes on BTA19, *GH1* and *GAA*, which are associated with production traits, and two candidate genes on BTA8, *U6* and *SLC44A1*, which are associated with NS and FSTC.

**Table 5:** Summary statistics of the effects of uniquely identified ROH with unfavorable effects on production and fertility traits

Trait	Minimum	Mean	Maximum	Standard deviation	−log_10_ (P value)
MY (kg)	− 410.65	− 247.30	− 114.57	76.20	3.22
FY (kg)	− 15.81	− 11.46	− 8.32	2.16	3.20
PY (kg)	− 16.12	− 8.13	− 3.43	2.54	3.37
NS	0.09	0.16	0.23	0.05	3.57
NRR	− 0.10	− 0.07	− 0.04	0.02	3.55
FSTC (day)	2.78	4.81	7.87	1.32	3.43

MY: milk yield; FY: fat yield; PY: protein yield; NS: number of services; NRR: 56-day non-return rate; FSTC: first service to conception

## Discussion

In this study, unfavorable unique ROH genotypes were identified and their effects on production and fertility traits were investigated. The justification for using an algorithm that identified unfavorable unique ROH genotypes was based on previous studies that investigated the effect of a region present in a ROH on a phenotype of interest [19, 34]. However, in these studies, it was assumed that any ROH genotype within a region of interest carries an unfavorable effect. Alternatively, it is most probable that the unfavorable effect is caused by a single unique ROH genotype while the other ROH in the region of interest show no unfavorable effect. Consequently, identifying unique ROH genotypes with an unfavorable effect on phenotypes affords the opportunity to better manage the region across time.

To limit the number of spuriously identified ROH genotypes with unfavorable effects, stringent criteria that only retained a minimum of 50 SNPs within a ROH region and a significant threshold determined by an FDR of 1% were applied. Overall, 133 and 34 ROH genotypes (see Additional file 2: Table S2 and Additional file 3: Table S3) associated with production and fertility traits were identified, respectively, with those for production traits having higher significance levels (i.e., lower *P values*). This is in line with previous studies [35, 36] and was expected given that fertility traits have a lower heritability and are largely influenced by environmental conditions and management decisions, as well as being difficult to measure [37]. For production traits, the estimated significant unfavorable effects ranged from − 114.57 to − 410.65 kg for MY, − 8.32 to − 15.81 kg for FY and − 3.43 to − 16.12 kg for PY (Table 5). These estimates are within the ranges reported by Martikainen et al. [38] for Finnish Ayrshire cattle, in which reductions ranging from − 140 to − 350 kg for MY, − 4 to − 16 kg for FY and − 5 to − 12 kg for PY were reported. The slightly higher estimates found in our study may result from the higher rate of inbreeding occurring in North America Holstein cattle [5]. Similarly, Pryce et al. [19] published a reduction in milk yield that ranged from − 161 to − 260 kg in Australian Holstein and Jersey cattle. Basically, this indicates that some ROH genotypes exhibit unfavorable effects on production traits and hence, selection programmes could benefit from controlling these ROH genotypes during mate allocation decisions.

For fertility, the average significant unfavorable effect was estimated to be 0.16 for NS, -0.07 for NRR and 4.81 days for FSTC. These averages are within the 0.12 to 0.31 range for the most significant ROH genotype reported by Martikainen et al. [38] for NS in Finnish Ayrshire heifers. These authors also reported an increase that ranged from 6.00 to 12.80 in the interval from first to last insemination (IFL), a trait similar to FSTC used in our study. In addition, the negative effects of ROH regions found in our study corroborate the results of previous studies that reported negative effects of ROH regions [19, 34], including in other species. For example, Howard et al. [28] observed that Landrace pigs that carry ROH regions on SSC9 (28.9–30.6 Mb) had a 4.0% higher chance of having stillbirths than pigs that did not carry the ROH.

Chromosomal ROH regions were further investigated to identify potential candidate genes that co-localize with unfavorably identified ROH genotypes associated with multiple traits. Notably, the largest number of ROH genotypes with unfavorable effects was on BTA19 for production traits and most of these were shared among production traits. Interestingly, the dwarfism growth-hormone deficiency gene (*GH1*) was identified in this region. The protein encoded by this gene is involved in the selective and non-covalent interaction with the growth hormone receptor. Qanbari et al. [39] identified the *GH1* gene using the extended haplotype homozygosity test in German Holsteins as a candidate gene for milk components in a study on signatures of selection, hence, selection could have a role in the increased homozygosity of this region. However, the specific objective of our study was to identify ROH genotypes with unfavorable effects on economically important traits. One possible explanation for identifying this genomic region could be genetic hitchhiking, whereby unfavorable alleles in linkage disequilibrium with favorable alleles are increased during selection. Furthermore, ROH regions on BTA14 (0.88–3.00 Mb) were identified with an unfavorable association with MY and FY, and these regions are 250 kb upstream of the *DGAT1* gene. In addition, in a study on Italian Holsteins, Minozzi et al. [36] identified SNPs within these regions with a negative effect on MY. Thus, such regions with harmful effects may be closely linked to a QTL region under selection. Similarly, regions located around the *DGAT1* gene have been identified to have a negative impact on cow mortality in US Holstein cows [40].

For fertility traits, a region on BTA1 spanning from 79.56 to 80.70 Mb that harboured 22 mapped genes was identified and found to have a significant unfavorable effect on NRR. In accordance with the study of Höglund et al. [41], this region was also identified as having a negative effect on NRR in Nordic dairy cattle. Similarly, Ben Jemaa et al. [42] identified the same region on BTA1 in French dairy cattle with a negative association with NRR. Furthermore, previously identified putative regions on BTA18 associated with fertility traits [35, 43] were also identified in our study. These findings provide evidence that the identified ROH genotypes strongly affect fertility in dairy cattle and co-localize with regions identified in previous studies.

Such unique ROH genotypes with an unfavorable association across multiple traits are more likely to be implicated in inbreeding depression, thereby reducing the overall performance of the individual carrying these regions. Thus, these identified unfavorable ROH genotypes could be incorporated into already existing algorithms designed to reduce the harmful effect of deleterious haplotypes in mating decisions [44, 45]. In addition, Howard et al. [28] generated an inbreeding load matrix (ILM) from the estimated effects of all identified unfavorable ROH genotypes as well as their associated probabilities for progeny of any given mating pair, thereby proposing that the diagonal of the ILM represents the functional inbreeding load of the individual (IIL). These authors found that the coefficient of the regression on IIL was closely related to progeny performance when compared to other genome-wide inbreeding measures.

## Conclusions

Unique ROH genotypes were identified with an unfavorable association within traits and across multiple traits. Furthermore, some of these regions were found to harbour potential candidate genes that co-localize with previously detected regions known to have negative associations with production and fertility traits. Therefore, controlling the occurrence of these identified unfavorable homozygous regions would be beneficial to prevent the adverse effect of inbreeding depression. In breeding programs, the algorithms for mate selection programs could be used to identify individuals that carry these unfavorable regions, and then to remove them from mating in order to minimise the frequency of the unfavorable ROH genotypes in future generations. Further research is warranted to refine and validate the identified ROH genotypes before implementation in selection programs.

## Supplementary Information

**Additional file 1: Table S1.** List of candidate genes and their position (ARS-UCD 1.2) located within the identified unique ROH that were found to be negatively associated with milk production and fertility traits in Canadian Holsteins

**Additional file 2: Table S2.** List of estimated effects of identified unique ROH genotypes with their chromosomal number, position, number of SNPs, P value and length for production traits

**Additional file 3: Table S3.** List of estimated effects of identified unique ROH genotypes with their chromosomal number, position, number of SNPs, P value and length for fertility traits

## Data Availability

All the necessary information needed to support the results of this paper are included within the article. Data that support the findings of this study are available from Lactanet- Canadian Dairy Network upon reasonable request.
